# Establishment of a serological molecular model for the early diagnosis and progression monitoring of bone metastasis in lung cancer

**DOI:** 10.1186/s12885-020-07046-2

**Published:** 2020-06-16

**Authors:** Xiaoyan Teng, Lirong Wei, Liming Han, Daliu Min, Yuzhen Du

**Affiliations:** 1 Department of Laboratory Medicine, Shanghai Jiao Tong University Affiliated Sixth People’s Hospital of Shanghai, Shanghai, 200233 P.R. China; 2 Department of Oncology, Shanghai Jiao Tong University Affiliated Sixth People’s Hospital of Shanghai, Shanghai, 200233 China

**Keywords:** Bone microenvironment cytokines, Bone turnover markers, Diagnostic risk factors, Bone metastasis, Lung cancer

## Abstract

**Background:**

The prognosis is very poor for lung cancer patients with bone metastasis. Unfortunately, a suitable method has yet to become available for the early diagnosis of bone metastasis in lung cancer patients. The present work describes an attempt to develop a novel model for the early identification of lung cancer patients with bone metastasis risk.

**Methods:**

As the test group, 205 primary lung cancer patients were recruited, of which 127 patients had bone metastasis; the other 78 patients without bone metastasis were set as the negative control. Additionally, 106 healthy volunteers were enrolled as the normal control. Serum levels of several cytokines in the bone microenvironment (CaN, OPG, PTHrP, and IL-6) and bone turnover markers (tP1NP, β-CTx) were detected in all samples by ECLIA or ELISA assay. Receiver operating characteristic (ROC) curves and multivariate analyses were performed to evaluate diagnostic abilities and to assess the attributable risk of bone metastasis for each of these indicators; the diagnostic model was established via logistic regression analysis. The prospective validation group consisted of 44 patients with stage IV primary lung cancer on whom a follow-up of at least 2 years was conducted, during which serum bone biochemical marker concentrations were monitored.

**Results:**

The serological molecular model for the diagnosis of bone metastasis was logit (p). ROC analysis showed that when logit (p) > 0.452, the area under curve of the model was 0.939 (sensitivity: 85.8%, specificity: 89.7%). Model validation demonstrated accuracy with a high degree of consistency (specificity: 85.7%, specificity: 87.5%, Kappa: 0.770). The average predictive time for bone metastasis occurrence of the model was 9.46 months earlier than that of the bone scan diagnosis. Serum OPG, PTHrP, tP1NP, β-CTx, and the diagnostic model logit (p) were all positively correlated with bone metastasis progression (*P* < 0.05).

**Conclusions:**

This diagnostic model has the potential to be a simple, non-invasive, and sensitive tool for diagnosing the occurrence and monitoring the progression of bone metastasis in patients with lung cancer.

## Background

Lung cancer patients are prone to developing bone metastasis accompanied by bone-related events such as bone pain, fracture, hypercalcemia, and nerve compression, which seriously affect patient quality of life [1, 2]. Clinically, the diagnosis of bone metastasis from lung cancer relies mainly on clinical symptoms and bone scans. There is no effective way to control bone destruction when tumour metastasis results in radiographically detectable lesions [3]. Therefore, the establishment of a non-invasive method for the early diagnosis of bone metastasis could significantly improve the prognosis and quality of life of patients with lung cancer. When bone metastasis occurs, osteoblasts in the bone microenvironment (BME) secrete calcineurin (CaN) and osteoprotegerin (OPG) [4, 5], and tumour cells secrete parathyroid hormone-related peptide (PTHrP) and interleukin- 6 (IL-6) [6, 7]. These cytokines interact with tumour cells, osteoblasts, osteoclasts, and stromal cells in the BME to promote osteogenic differentiation and bone resorption. When the delicate balance of bone metabolism is broken, bone turnover markers in the blood, including the bone formation marker tP1NP and the bone resorption marker β-CTx, are significantly increased [8, 9]. BME cytokines (CaN, OPG, PTHrP, and IL-6) and bone turnover markers (tP1NP and β-CTx) can be detected in the serum of patients [10, 11]. These serum indicators can serve as special serum bone biochemical markers for bone metastasis. Therefore, changes in the concentrations of these bone biochemical markers may be associated with the progression of tumour bone metastasis.

In this study, the serum concentrations of BME cytokines (CaN, OPG, PTHrP, and IL-6) and bone turnover markers (tP1NP and β-CTx) were analysed in 205 lung cancer patients, and these bone biochemical markers were comprehensively analysed in relation to bone metastasis in lung cancer. The diagnostic values were assessed and a serological molecular model was established to provide an experimental basis for the early diagnosis and progression monitoring of lung cancer bone metastasis.

## Methods

### Patients’ clinical characteristics

This study was approved by the Ethics Committee of the East Campus of Shanghai Sixth People’s Hospital, affiliated with Shanghai Jiaotong University. All participants in the study provided written informed consent prior to recruitment.

Patients with stage IV primary lung cancer that were admitted to the East Campus of Shanghai Sixth People’s Hospital from October 2015 to August 2016 were enrolled. After excluding patients who had received chemotherapy, radiotherapy, immunotherapy, bisphosphonate therapy, or other treatment within 3 months prior enrolment and those with endocrine disorders or immune or metabolic diseases, a total of 205 patients were enrolled in the study. Follow-up was conducted until October 2018, with a minimum follow-up period of 2 years. Of the enrolled patients, 127 (bone metastasis group) had bone metastasis, and the other 78 (non-bone metastasis group) did not have bone metastasis, as determined on the basis of pathology or bone scans. Bone scan examination was performed every 6 months to monitor the progression of bone metastasis in all patients. In addition, 106 healthy volunteers were recruited as normal controls. The control group was age- and gender-matched with the test group. The clinical characteristics of the patients and healthy volunteers are shown in Supplementary Table 1.

The prospective validation study included 44 patients with stage IV primary lung cancer who underwent a complete surgical resection between October 2015 and August 2016 and were followed until October 2018, for a minimum of 2 years. All patients had been diagnosed with non-bone metastasis by pathology or bone scan at the initial presentation. Bone scan examination was performed every 6 months to monitor bone metastasis in all patients. The clinical characteristics of 44 patients, including age, gender, histological type, and stage are shown in Supplementary Table 2. Changes in the serum levels of bone biochemical markers were monitored during follow-up. Over the follow-up period, bone metastasis occurred in some patients, and serum samples obtained at the time of bone metastasis diagnosis were used as endpoint follow-up samples. For the patients without bone metastasis, the serum obtained after 2 years of follow-up was used as the endpoint follow-up sample.

### Collection and determination of the serum samples

A total of 2–4 ml of fasting venous blood from patients and healthy volunteers was collected in the morning and left at 25 °C for 30 min. The supernatant serum was obtained by centrifugation at 4000 *g* for 10 min, and then the serum samples from the individuals were transferred to new tubes in 500 μl aliquots and stored at − 80 °C.

The serum concentrations of tP1NP (03141071190), β-CTx (11972308122), and IL-6 (05109442190) were quantified using a Diagnostics Cobas E601 fully automated electrochemiluminescence immunoassay (ECLIA) (Swiss Roche) and its original reagents. The inter-assay precision CVs of tP1NP, β-CTx, and IL-6 were less than 3.2, 4.7, and 3.9%, respectively. The intra-assay precision CVs of tP1NP, β-CTx and IL-6 were less than 8.7, 5.7, and 5.1%, respectively.

The serum concentrations of CaN (BYS10821B), OPG (BYS10849B), and PTHRP (BYS10753B) were measured quantitatively by an enzyme-linked immunosorbent assay (ELISA) kit (Shanghai Boyan Biotech, China). All of the methods were performed in accordance with the manufacturer’s instructions. The optical densities (OD) at 450 nm and 622 nm were detected with a microplate reader (Biotech, Winooski, VT, USA), and the repeatability CV was less than 15%.

### Statistical analysis

MedCalc software (Medcalc, Mariakerke, Belgium) and GraphPad Prism (San Diego, CA, USA) were used for statistical analysis and mapping. The serum levels of bone biochemical markers of the two groups were compared with the nonparametric Kruskal Wallis test. Spearman correlation analysis was used to assess the correlation between the serum concentrations of these indicators. An ROC curve analysis was used to evaluate the diagnostic value of each of these indicators for bone metastasis in lung cancer. The optimal Youden index method was used to determine the cut-off value of these indicators for the diagnosis of bone metastasis. In addition, univariate and multivariate logistic regression analyses were performed to assess the risk attributed by each of these markers for the development of bone metastasis in lung cancer and to establish a serological molecular model for the diagnosis of bone metastasis in lung cancer. The Hosmer-Lemeshow test and an ROC curve analysis were used to evaluate the model-fitting effect and diagnostic accuracy, and the Kappa test was used to evaluate the consistency of the follow-up bone scan diagnosis results with the model diagnosis results. In the follow-up study, the change in serum index was calculated with the following equation: [% year change (median)] = [(follow-up level - initial level) / (year * initial level)]. The nonparametric Mann-Whitney test was used to compare the initial levels, follow-up levels, % year change, and logit (p) between the bone metastasis group and the non-bone metastasis group. The nonparametric Wilcoxon test was used to compare differences within the bone metastasis group and the non-bone metastasis group, including initial levels, follow-up levels, and model logit (p). A value of *P* < 0.05 was considered statistically significant.

### Clinical practice points

1. Patients with lung cancer have high serum levels of bone biochemical markers (BME cytokines and bone turnover markers) and are prone to bone metastasis; there is no existing method that can diagnose bone metastasis early or monitor its occurrence in lung cancer patients.
2. For patients with stage IV lung cancer, those with high serum concentrations of BME cytokines and bone turnover markers were significantly associated with the occurrence of bone metastasis.
3. In addition to clinical pathological factors, this serum bone biochemical marker model could be a simple, non-invasive, and sensitive tool for the diagnosis and progression monitoring of bone metastasis in patients with lung cancer.

## Results

### Serum levels of BME cytokines and bone turnover markers in lung cancer patients with or without bone metastasis disease

**Fig. 1:**
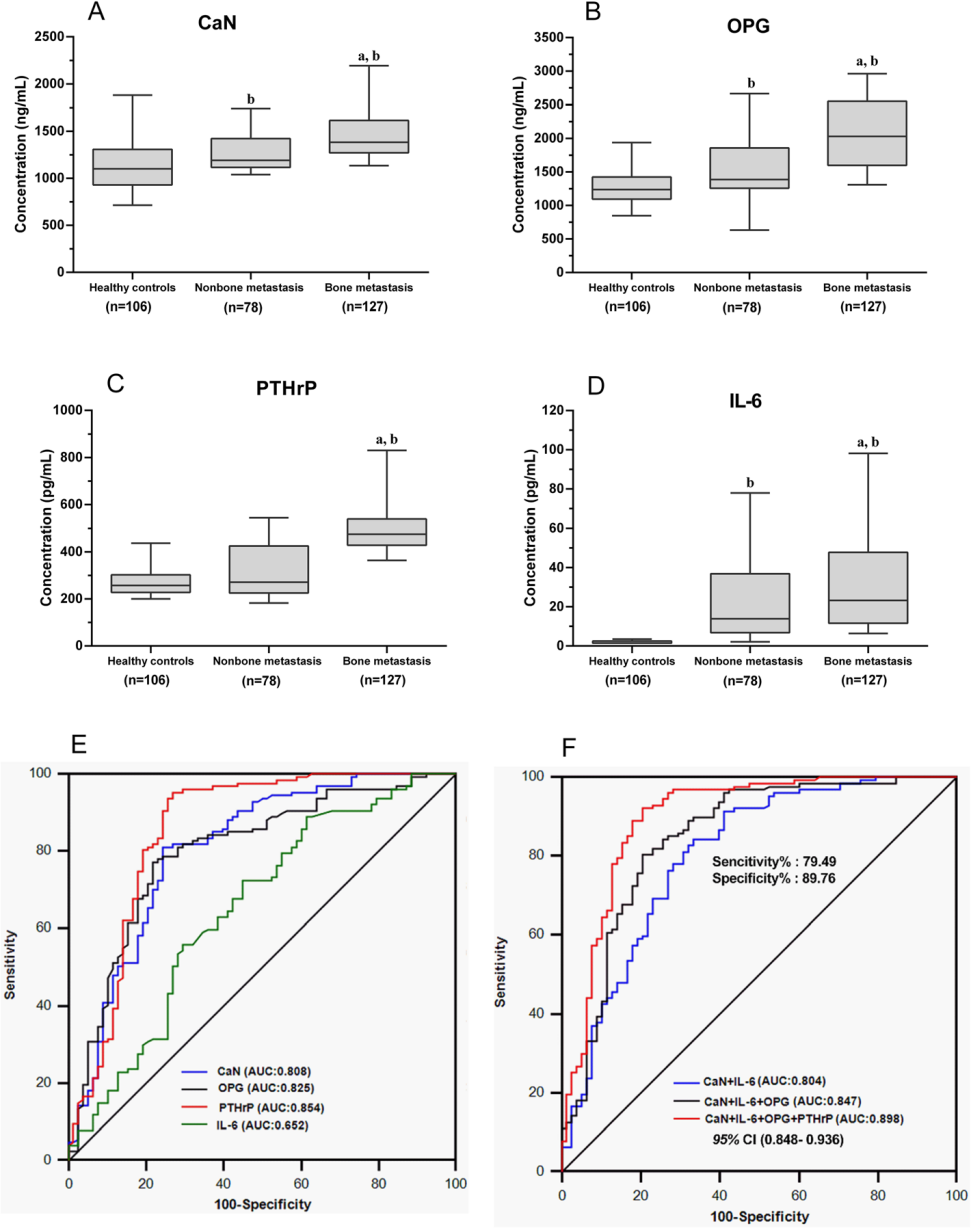
Serum levels of BME cytokines and their diagnostic abilities for bone metastasis in stage IV lung cancer patients. **a**, **b**, **c**, **d** Box plots of the serum levels of the BME cytokines CaN, OPG, PTHrP, and IL-6 in three groups, including the bone metastasis group (*n* = 127), the non-bone metastasis group (*n* = 78), and the healthy control group (*n* = 106). From the bottom up, the boxes indicate the 25th, 50th (median), and 75th percentiles, while the bars indicate the 10th and 90th percentiles, respectively. Significance levels were obtained from the nonparametric Kolmogorov-Smirnov test: (**a**) *P* < 0.05: vs non-bone metastasis, (**b**) *P* < 0.05: vs healthy controls. **e** ROC analysis of the diagnostic abilities of serum bone microenvironment cytokines for bone metastasis. **f** ROC analysis of the diagnostic efficiencies of different combinations of the bone microenvironment cytokines for bone metastasis

Compared with the healthy control, the serum concentrations of BME cytokines and bone turnover markers were significantly higher in the lung cancer bone metastasis group (*P* < 0.05). In non-bone the metastasis group, serum levels of all serum bone biochemical markers were slightly higher than those of the healthy controls (*P* < 0.05) (Fig. 1 A/B/D), except for PTHrP (*P* > 0.05) (Fig. 1 C).

**Fig. 2:**
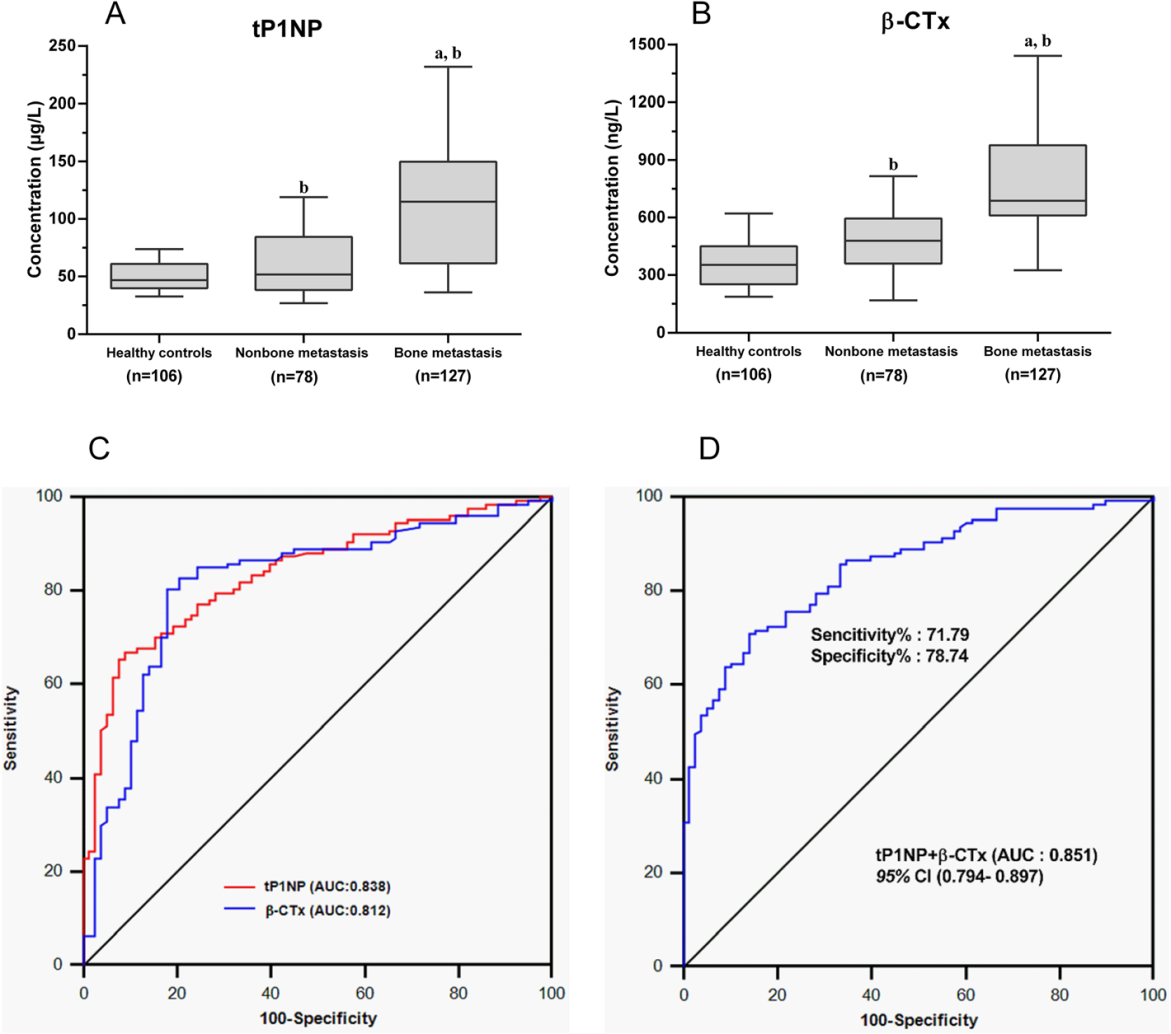
Serum levels of bone turnover markers and their abilities to diagnose bone metastasis in stage IV lung cancer patients. **a**, **b** Box plot distribution of the serum levels of the bone turnover markers tPINP and β-CTx from three groups, including the bone metastasis group (n = 127), the non-bone metastasis group (n = 78), and the healthy control group (n = 106). For details of the box plots, see the legend of Fig. 1. **c** ROC analysis of the ability of the serum bone turnover markers tP1NP and β-CTx to diagnose bone metastasis. **d** ROC analysis of the diagnostic efficiency of the combination of tP1NP and β-CTx

Compared with those in the non-bone metastasis group, the serum levels of all BME cytokines in the bone metastasis group were significantly elevated, including CaN, OPG, PTHrP, and IL-6 (*P* < 0.05) (Fig. 1 A/B/C/D). Similar results were observed for the bone turnover markers. The serum levels of tP1NP and β-CTx in the bone metastasis group were significantly higher than those in the non-bone metastasis group (*P* < 0.05) (Fig. 2 A/B).

### Diagnostic values of serum BME cytokines and bone turnover markers for bone metastasis in patients with lung cancer

The ROC curve analysis showed that the serum BME cytokines CaN, OPG, and PTHrP had high diagnostic values for bone metastasis in lung cancer. The areas under curve (AUC) for these indicators were 0.808, 0.825, and 0.854, and the cut-off values were 1257.80 ng/ml, 1547.00 ng/ml, and 322.30 pg/ml, respectively. The diagnostic ability of serum IL-6 was limited; the AUC was 0.652, and the cut-off value was 14.36 pg/ml. The AUC for the four cytokines combined (CaN, OPG, PTHrP, and IL-6) was 0.898 [*P* < 0.001, 95% CI (0.848–0.936)], with a sensitivity of 79.49% and a specificity of 89.76% (Fig. 1E/F, Tables 1 and 2). The diagnostic ability of the cytokine combination was superior to that of each cytokine alone.

**Table 1:** Diagnostic ability of serum bone biomarkers to distinguish lung cancer patients with bone metastasis from those without bone involvement

			Cut-off	Sensitivity	Specificity		Diagnostic
Marker	AUC	95% CI	level	%	%	*P value*	accuracy (%)
BME cytokine							
CaN (ng/mL)	0.81	0.747–0.859	1257.80	81.10	75.60	< 0.001	78.74
OPG (ng/mL)	0.83	0.760–0.856	1547.00	77.20	78.20	< 0.001	77.30
PTHrP (pg/mL)	0.85	0.798–0.899	322.30	95.30	73.10	< 0.001	51.70
IL-6 (pg/mL)	0.65	0.583–0.717	14.36	72.40	55.10	< 0.001	65.90
Bone turnover marker							
tP1NP (μg/L)	0.84	0.780–0.885	93.59	66.90	91.00	< 0.001	74.71
β-CTx (ng/L)	0.81	0.749–0.865	619.70	80.30	82.10	< 0.001	83.91

*AUC* Area under the curve, *CI* Confidence interval

**Table 2:** Diagnostic efficiency of different combinations of serum bone biomarkers to distinguish lung cancer patients with bone metastasis from those without bone involvement

			Sensitivity	Specificity		Diagnostic
Combination	AUC	95% CI	%	%	*P* value	accuracy (%)
BME cytokines						
CaN+IL-6	0.804	0.743–0.856	60.26	84.25	< 0.001	75.12
CaN+IL-6 + OPG	0.847	0.790–0.893	74.36	84.25	< 0.001	80.49
CaN+IL-6 + OPG + PTHrP	0.898	0.848–0.936	79.49	89.76	< 0.001	85.63
Bone turnover markers						
tP1NP + β-CTx	0.851	0.794–0.897	71.79	78.74	< 0.001	76.1
Independent diagnostic risk factors						
OPG + PTHrP	0.893	0.842–0.931	78.21	90.55	< 0.001	76.1
OPG + PTHrP+tP1NP	0.924	0.864–0.952	81.69	84.47	< 0.001	83.91
OPG + PTHrP+tP1NP + β-CTx	0.94	0.898–0.968	84.62	88.98	< 0.001	87.32

The serum bone turnover markers tP1NP and β-CTx also had high diagnostic values for bone metastasis in lung cancer. The AUCs of tP1NP and β-CTx were 0.838 and 0.812, and the cut-off values were 93.59 μg/L and 619.70 ng/ml, respectively. The AUC of tP1NP combined with β-CTx was 0.851 [*P* < 0.001, 95% CI (0.794–0.897)], the sensitivity was 71.79%, and the specificity was 78.74% (Fig. 2 C/D, Tables 1 and 2).

The combination of bone turnover markers showed stronger diagnostic performance than each one used alone, but the sensitivity was limited (Tables 1 and 2).

### Correlation between serum levels of BME cytokines and bone turnover markers

**Fig. 3:**
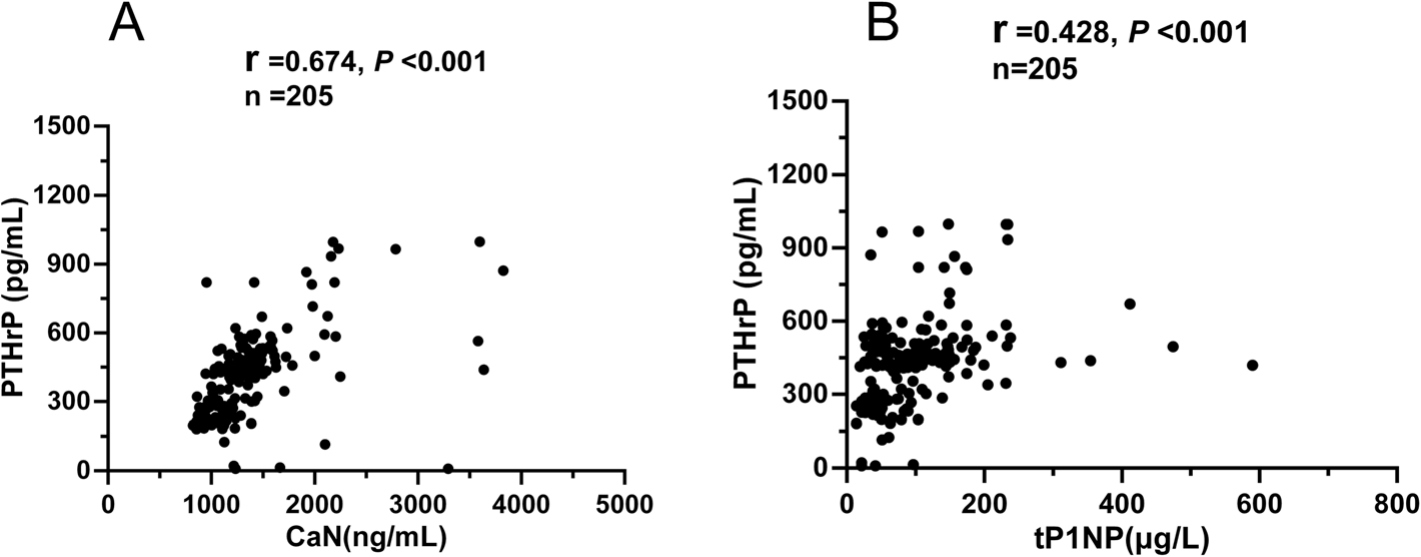
Correlations between serum levels for BME cytokines and bone turnover markers in 205 patients with stage IV primary lung cancer. **a** Correlation between serum levels of the bone microenvironment cytokines PTHrP and CaN. **b** Correlation between serum levels of the bone microenvironment cytokine PTHrP and bone turnover marker tP1NP

Serum BME cytokines and bone turnover markers were analysed in 205 patients with stage IV primary lung cancer. A positive correlation was found between the four cytokines (r values ranged from 0.289 to 0.674, *P* < 0.001) (Supplementary Table 3), especially between CaN and PTHrP (r = 0.674, *P* < 0.001) (Fig. 3A). Interestingly, there was a positive correlation between PTHrP and tP1NP (r = 0.428, *P* < 0.001) (Fig. 3B).

### Establishment and validation of a serological molecular model for the diagnosis of bone metastasis in lung cancer

**Fig. 4:**
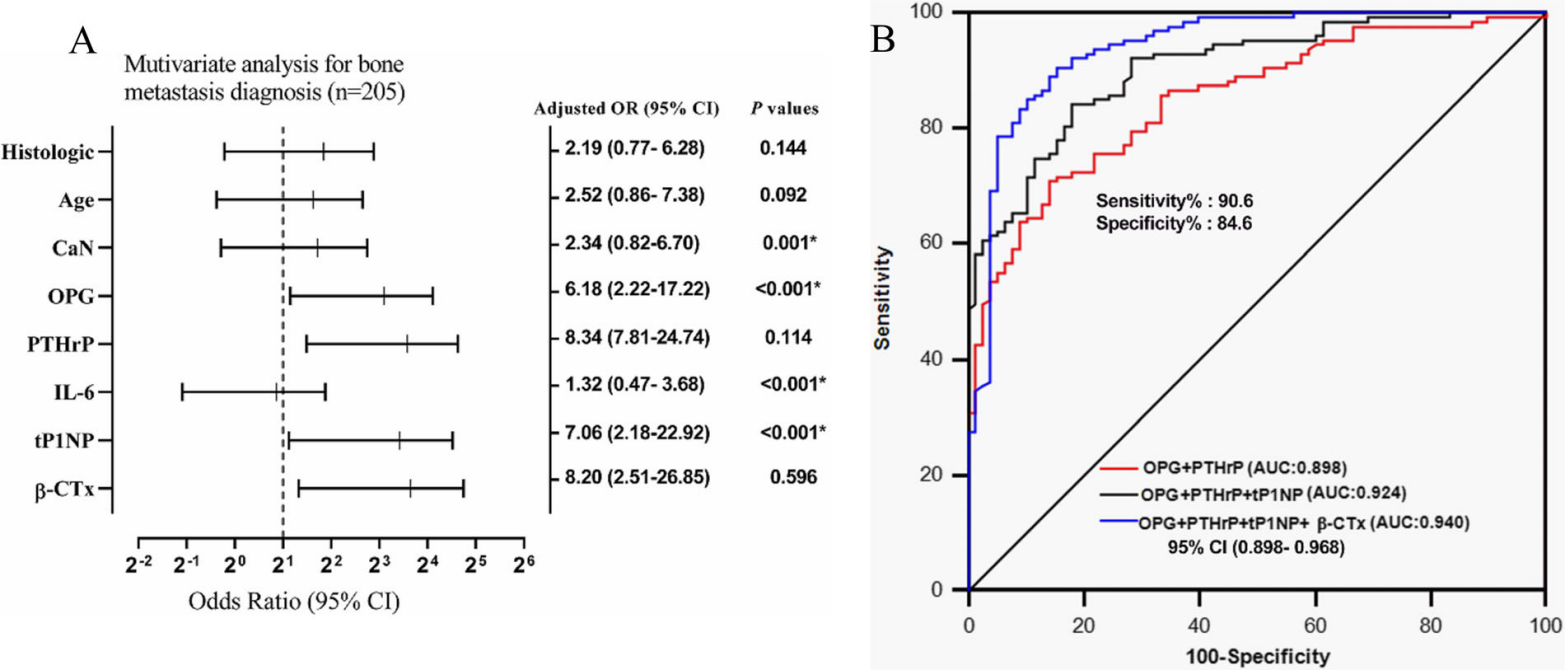
Evaluation of the attributable risk and diagnostic efficiency of these parameters for bone metastasis in 205 patients with stage IV primary lung cancer. **a** Multivariate logistic regression analysis to evaluate the attributable risk of these parameters for bone metastasis. OR values were presented by log2 transformation. OR: odds ratio, CI: confidence interval. * *P* < 0.05 was considered statistically significant. **b** ROC analysis to assess the diagnostic efficiency for bone metastasis of different combinations of the four independent diagnostic risk factors, including BME cytokines (OPG and PTHrP) and bone turnover markers (tP1NP and β-CTx)

Inclusive indicators included the serum levels of BME cytokines and bone turnover markers, age, sex, pathological type, stage, and bone scan results of the patients (see Supplementary Table 4 for details). The patients were grouped according to cut-off values and subjected to univariate and multivariate regression analyses. Serum CaN, OPG, PTHrP, IL-6, tP1NP, β-CTx, and tissue type were the univariate diagnostic factors for bone metastasis in lung cancer (*P* < 0.05); age and sex were the only two excluded factors. All variables with *P* < 0.05 in the univariate analysis were analysed by multivariate logistic regression to determine whether these serum markers were independently related to the diagnosis of bone metastasis in lung cancer. Multivariate logistic regression analysis indicated that only OPG (*P* < 0.001), PTHrP (*P* < 0.001), tP1NP (*P* = 0.001), and β-CTx (*P* < 0.001) were independent diagnostic factors for bone metastasis in lung cancer, while CaN and IL-6 were not (Fig. 4A and Supplementary Table 4).

**Fig. 5:**
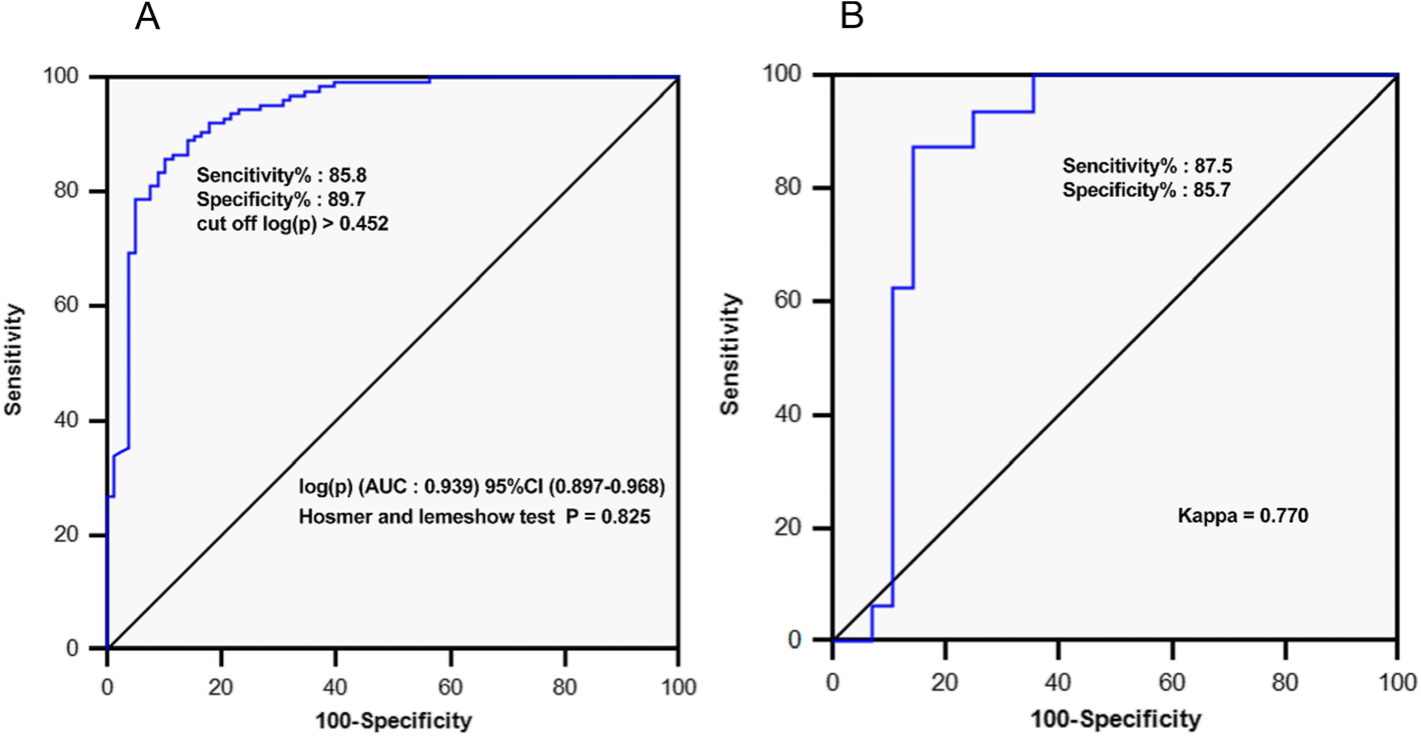
Diagnostic efficiency of the diagnostic model for bone metastasis in patients with stage IV primary lung cancer. **a** Evaluation of the model fitting degree and diagnostic accuracy of the model. **b** ROC analysis of consistency and effectiveness in the diagnosis of bone metastasis of the model

ROC analysis showed that the combination of four independent diagnostic factors, OPG, PTHrP, tP1NP, and β-CTx, had the highest diagnostic efficiency, with a diagnostic accuracy of 87.32%. The AUC of this combination was 0.940 [*P* < 0.001, 95% CI (0.898–0.968)] (Fig. 4B and Table 2). Multivariate logistic regression analysis established a molecular model for bone metastasis diagnosis: logit (p) = − 8.4988 + 0.0211 * tP1NP + 0.0079 * PTHrP + 0.0016* OPG + 0.0021*β-CTx. The Hosmer-Lemeshow test showed that *P* = 0.825, which indicated a good model fit. When logit (p) > 0.452, the sensitivity and specificity of the predictive model were 85.8 and 89.7%, respectively (Fig. 5A).

**Fig. 6:**
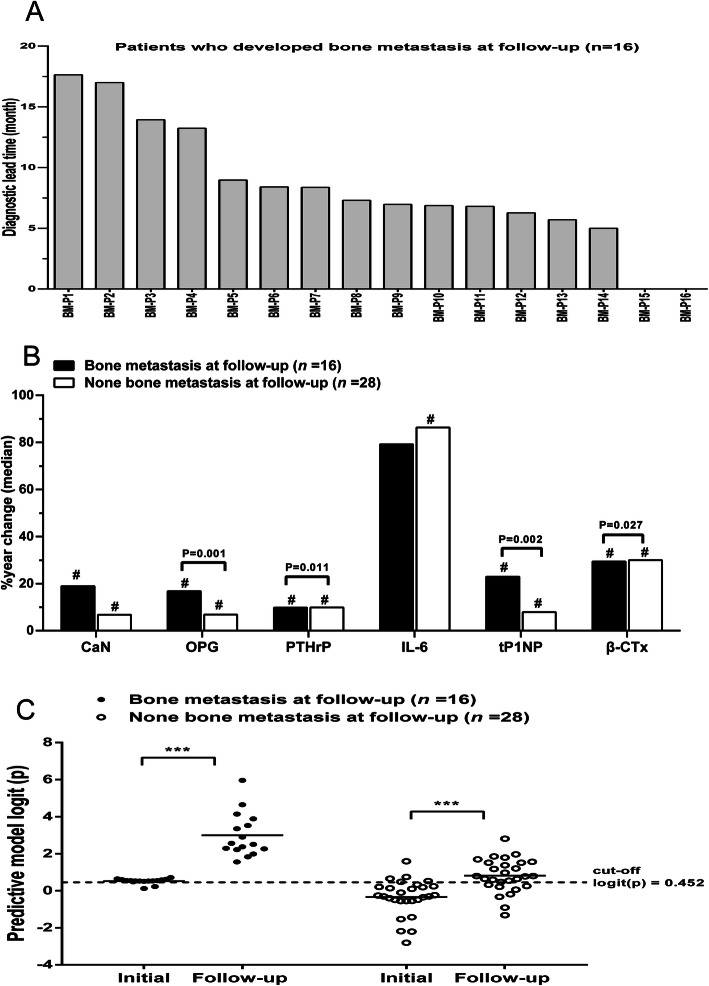
Evaluation of the progression monitoring abilities of the serum levels of bone biochemical markers and the diagnostic model. **a** The lead time of the diagnostic model of bone metastasis was 5.00–17.63 months, compared with the date of bone scan diagnosis for 16 patients with stage IV lung cancer who developed bone metastasis during follow-up. **b** The changes in the serum levels of bone biochemical markers in patients with lung cancer who developed or remained free of bone metastasis during follow-up. For each patient, the changes in the serum levels of bone biochemical markers during follow-up were calculated as [% year change (median)] = [(follow-up level − initial level) / (2 * initial level)]. The bars represent the % year changes (median). The actual *P* value indicated in the figure refers to a comparison of the % year changes between the two groups. The # indicated in the figure represents *P* < 0.05: comparison between the follow-up levels and the initial levels within each group separately. **c** The diagnostic model logit (p) of the initial levels and follow-up levels of patients with lung cancer who developed or remained free of bone metastasis during follow-up. The horizontal line on the figure represents the median of the scatter plots for this group. ***P < 0.001, comparison between the follow-up levels and the initial levels in each group separately

To verify the accuracy and effectiveness of the predictive model for bone metastasis, 44 patients with stage IV primary lung cancer without bone metastasis were predicted by the model and followed up for at least 2 years. During the follow-up period, serum levels of OPG, PTHrP, tP1NP, and β-CTx were continuously monitored, along with the results of bone scans. Of the 44 patients, based on the model, 18 patients had high scores and were predicted to develop bone metastasis, and 26 patients were predicted not to develop the disease. Follow-up results showed that 16 patients developed bone metastasis. The specificity and sensitivity of the predictive model were 85.7 and 87.5%, respectively, and the Kappa value was 0.770, which showed that the predictive results of the model were highly consistent with the bone scan results (Fig. 5B), but the average predictive time of the serum model was 9.46 months earlier than that of the bone scan (Fig. 6A).

### The roles of BME cytokines, bone turnover markers, and diagnostic models in monitoring the progress of bone metastasis

During the follow-up period, the concentrations of BME cytokines and bone turnover markers in serum samples at the start and end of the follow-up period were measured and recorded as the initial and follow-up levels, respectively.

During the follow-up of 44 patients, 16 patients with lung cancer had bone metastasis (bone metastasis group). The follow-up levels of these serum indicators were significantly higher than their initial levels, except for IL-6 (Table 3). The other 28 patients without bone metastasis (non-bone metastasis group) showed only slight increases in the serum levels of these indicators (*P* < 0.05). The % year changes of serum OPG (*P* = 0.001), PTHrP (*P* = 0.011), tP1NP (*P* = 0.002), and β-CTx (*P* = 0.027) in patients with bone metastasis were significantly higher than those in patients without bone metastasis, but there was no significant difference in serum CaN or IL-6 (Fig. 6B). Therefore, the changes in serum concentrations of OPG, PTHrP, tP1NP, and β-CTx are related to the progression of bone metastasis in lung cancer patients.

**Table 3:** The initial levels of serum bone bimarkers in lung cancer patients who developed bone metastasis and the others who remained free of bone metastasis during follow-up

	Serum bone marker at initial level		
Marker	Yes (*n* = 16)	No (*n* = 28)	*P value*
BME cytokine			
CaN (ng/mL)	1263.54 (1713.01)	1287.27 (1418.43)	0.625
OPG (ng/mL)	1784.69 (2319.0)	1638.18 (1789.94)	0.105
PTHrP (pg/mL)	401.12 (476.32)	380.38 (431.93)	0.28
IL-6 (pg/mL)	23.73 (30.01)	23.29 (45.19)	0.136
Bone turnover marker			
tP1NP (μg/L)	80.83 (101.60)	71.06 (81.62)	0.11
β-CTx (ng/L)	610.0 (894.06)	489.58 (624.24)	0.081

Data are expressed as median (interquartile)

In addition, the diagnostic model logit (p) of patients with bone metastasis from lung cancer was significantly higher than that of lung cancer patients without bone metastasis (*P* < 0.001). In addition, the diagnostic logit (p) of bone metastasis in lung cancer patients increased over time (*P* < 0.001) (Fig. 6C).

## Discussion

BME cytokines and bone turnover markers are involved in the early survival mechanisms of tumour cells in bone tissue, such as colonization, dormancy, micrometastasis, and metastasis formation [12]. In this study, serum BME cytokines (CaN, OPG, PTHrP, and IL-6) and bone turnover markers (tP1NP and β-CTx) were measured in lung cancer patients. The concentrations of these markers in the bone metastasis group were significantly higher than those in the non-bone metastasis group and the healthy control group. An ROC curve analysis showed that serum CaN, OPG, PTHRP, tP1NP, and β-CTx were valuable in the diagnosis of bone metastasis in lung cancer. Univariate and multivariate logistic regression analyses showed that serum OPG, PTHrP, tP1NP, and β-CTx were independent diagnostic factors for bone metastasis in lung cancer. Therefore, a serum molecular model for the diagnosis of bone metastasis in lung cancer was established. Forty-four patients with lung cancer were followed up to verify the diagnostic value of this serum model for bone metastasis in lung cancer. It was found that the model could accurately indicate the occurrence of bone metastasis in lung cancer, and the diagnosis time of this model for bone metastasis was 9.46 (5.00–17.63) months earlier than that of bone scan examination. In addition, the results also suggested that serum OPG, PTHrP, tP1NP, and β-CTx along with the serological molecular model could be used to monitor the progression of bone metastasis in lung cancer.

Previous studies have rarely reported the correlation between serum BME cytokine levels and bone metastasis in lung cancer. At present, there have been no reports on the application of BME cytokines combined with bone turnover markers in the diagnosis of bone metastasis in lung cancer. In a previous study, Zhou had established a molecular model which involved using four markers to perform immunohistochemical analysis on 105 stage III lung cancer patients, and the model was prospectively validated. It exhibited a prediction sensitivity of 85.7% and a specificity of 66.7% [13]. However, the acquisition of tissue samples was limited and could not be monitored continuously or dynamically. In this study, a serum molecular diagnostic model for bone metastasis in lung cancer was established for the first time by combining BME cytokines and bone turnover markers.

Bone biochemical markers that can be observed during bone metastasis include secretory cytokines in the BME, the collagen degradation product tP1NP and β-CTx, which are formed during bone resorption and bone formation. BME cytokines include CaN, OPG, PTHrP, and IL-6. CaN is a key regulator of gene transcription and differentiation in osteoclasts, and it can promote the differentiation and maturation of osteoclasts through the Ca-CaN-NFATc1 signalling pathway [14]. OPG, a soluble tumour necrosis factor receptor (TNFR), can inhibit the apoptosis of tumour cells by blocking the apoptotic pathway of TRAIL, thereby promoting their survival and growth [15]. On the other hand, OPG can inhibit the function and formation of osteoclasts by inhibiting the RANKL signalling pathway [16]. In addition, OPG plays a key role in promoting angiogenesis [17]. The cytokine IL-6 is a polypeptide with multifunctional biological activity. IL-6 can regulate the balance between bone formation and bone resorption [18]. This study showed that the serum level of CaN had a high diagnostic value for bone metastasis in lung cancer, which is consistent with the recent report that the serum CaN level has a high positive correlation with bone metastasis in breast cancer patients [19]. In prostate, breast, and lung cancer, serum OPG levels of bone metastasis patients tend to be higher than those of non-bone metastasis patients [20–22]. The multitude of biological effects of IL-6 produced more interference factors in the diagnosis and monitoring of bone metastasis, so the serum level of IL-6 had no diagnostic value [7, 18]. Serum PTHrP levels had a good application value in the early diagnosis and progression monitoring of bone metastasis in lung cancer, and the combined diagnostic value of OPG and PTHrP was higher. Previous studies have found that high PTHrP levels are significantly associated with high bone metastasis rates and low median survival rates in patients with lung cancer, which is consistent with our results [23–25].

The bone turnover markers tP1NP and β-CTx are products of bone metabolism, and they are important serum biochemical markers of bone formation and bone resorption, respectively [26, 27]. In the process of bone metastasis, once the balance of bone formation and bone absorption has been destroyed, the concentrations of tP1NP and β-CTx in the blood will increase significantly [28–30]. The present study suggests that serum levels of tP1NP and β-CTx can be used for the prediction and progression monitoring of bone metastasis in lung cancer.

The correlation analysis of cytokines showed a strong positive correlation between CaN and PTHRP (r = 0.674, *P* < 0.001) (Fig. 3A), indicating that in the BME, tumour cells interact with other cells through BME cytokines. Interestingly, the cytokine PTHrP also showed a moderately positive correlation with the bone turnover marker tP1NP (r = 0.428, *P* < 0.001) (Fig. 3B). The correlation between BME cytokines and bone turnover markers suggests that the combination of these two factors may be an effective method for improving the early diagnosis of bone metastasis. However, serum bone biochemical markers are susceptible to individual differences and seasonal variations [31, 32]. Therefore, a combination of these bone biochemical markers and bone scan techniques may be more conducive to the diagnosis of bone metastasis in lung cancer.

Multivariate logistic regression analysis revealed that the serum BME cytokines OPG and PTHrP and the bone turnover markers tP1NP and β-CTx were independent diagnostic factors for bone metastasis. Furthermore, a mathematical model was established to combine these indicators, so as to predict the risk of bone metastasis in patients with advanced lung cancer more accurately: logit (p) = − 8.4988 + 0.0211 * tP1NP + 0.0079 * PTHrP + 0.0016* OPG + 0.0021*β-CTx. The AUC of this model reached as high as 0.940. When logit (p) > 0.452, the specificity and sensitivity of bone metastasis diagnosis in lung cancer are 85.8 and 89.7%. Prospective verification of this model revealed that its sensitivity and specificity for the diagnosis of bone metastasis in lung cancer were 87.5 and 85.7%, respectively, and the Kappa value was 0.770, which was highly consistent with the actual results. A follow-up study of 44 patients for a minimum of 2 years confirmed that the model could accurately predict the occurrence of bone metastasis in lung cancer, and the prediction of bone metastasis was 9.46 (5.00–17.63) months earlier than medical imaging evidence appeared (Fig. 6A). Additionally, it was found that changes in serum OPG, PTHrP, tP1NP, β-CTx, and the diagnostic model logit (p) were positively correlated with the progression of bone metastasis, suggesting that the diagnostic model could be used to monitor bone metastasis progression. These results further prove the validity of the diagnostic model.

While these results present novel and innovative diagnostic solutions, the limitations of this study warrant clear recognition. The molecular model was established to predict the risk of bone metastasis in resected stage IV lung cancer in order to screen the patients with a high risk of bone metastasis for early intervention. Therefore, the method might be more suitable for predicting the risk of bone metastasis in stage IV patients than those in other stages before medical imaging evidence appears. In the future, the diagnostic value and prognostic value of this model will require validation through multicentre studies with larger sample sizes and retrospective or prospective studies at different disease stages, so as to improve the efficacy of this lung cancer bone metastasis diagnostic model.

## Conclusion

The serum molecular diagnostic model composed of BME cytokines (OPG and PTHrP) and bone turnover markers (tP1NP and β-CTx) can assist with the diagnosis and progression monitoring of bone metastasis in lung cancer. These bone biomarkers can be continuously (once every three to six months, depending on the patient’s situation) detected in the sera of lung cancer patients during disease progression, and a continuous, dynamic assessment of bone metastasis risk can be conducted based on the prediction model. If bone metastasis can be predicted early enough, then effective preventative measures could be taken, which may improve the life quality of the lung cancer patients. This model may provide a simple, non-invasive, and reliable method for the early screening of bone metastases in lung cancer patients.

## Supplementary information

**Additional file 1: Supplementary Table 1.** The clinical characteristics of 205 lung cancer patients for establishment of the diagnostic model of bone metastasis analysis.

**Additional file 2: Supplementary Table 2.** The basic information of 44 lung cancer patients for prospective validation analysis.

**Additional file 3: Supplementary Table 3.** Correlations of serum levels for BME cytokines and bone turnover markers in 205 patients with primary lung cancer stage IV.

**Additional file 4: Supplementary Table 4.** Univariate and multivariate analysis of diagnostic risk factors associated with bone metastasis diagnosis in 205 patients with lung cancer.

## Data Availability

Please contact the corresponding author for data request.

## Abbreviations

BME: Bone microenvironment
CaN: Calcineurin
OPG: Osteoprotegerin
PTHrP: Parathyroid hormone related-peptide
IL-6: Interleukin-6
tP1NP: Total amino-terminal propeptide of type I procollagen
β-CTx: Carboxy-terminal crosslinked telopeptide of type I collagen β isomer
AUC: Area under the curve
ROC: Receiver operating characteristics
CI: Confidence interval
OR: Odds ratio
