# Supplementary material for: Establishment of a serological molecular model for the early diagnosis and progression monitoring of bone metastasis in lung cancer
Source: BMC Cancer. 2020 Jun 16;20:562. doi: 10.1186/s12885-020-07046-2 (PMC7298761; doi:10.1186/s12885-020-07046-2)
Supplement: Supplementary file 1 — Additional file 1: Supplementary Table 1. The clinical characteristics of 205 lung cancer patients for establishment of the diagnostic model of bone metastasis analysis. [file 12885_2020_7046_MOESM1_ESM.docx]

|  | Total number of | Bone metastasis | Nonbone metastasis |  |
| --- | --- | --- | --- | --- |
| Characteristic | Patients (n)% | group (n)% | group (n) % | *P value* |
| Age (years) | 65.31 ± 0.68 | 64.03 ± 0.72 | 66.06 ± 0.77 | 0.26 |
| Histological type (n) |  |  |  | 0.34 |
| Adenocarcinoma | 132 (64.4) | 92 (72.4) | 35 (44.87) |  |
| Squamous cell carcinoma | 41 (20.0) | 20 (15.8) | 22 (28.21) |  |
| Small cell carcinoma | 32 (15.6) | 15 (11.8) | 21 (26.92) |  |
| Gender (n) |  |  |  | 0.21 |
| Men | 145 (70.7) | 86 (67.7) | 57 (73.08) |  |
| Women | 60 (29.3) | 41 (32.3) | 21 (26.92) |  |
| Stage Ⅳ (n) | 205 (100.0) | 127 (100.0) | 78 (100.0) |  |
| Total (n) | 205 (100.0) | 127 (100.0) | 78 (100.0) |  |
| *P* value: comparison between bone metastasis group and nonbone metastasis group. | | | | |

**Supplementary Table 1** The clinical characteristics of 205 lung cancer patients for establishment of the diagnostic model of bone metastasis analysis.
