# Supplementary material for: Establishment of a serological molecular model for the early diagnosis and progression monitoring of bone metastasis in lung cancer
Source: BMC Cancer. 2020 Jun 16;20:562. doi: 10.1186/s12885-020-07046-2 (PMC7298761; doi:10.1186/s12885-020-07046-2)
Supplement: Supplementary file 2 — Additional file 2: Supplementary Table 2. The basic information of 44 lung cancer patients for prospective validation analysis. [file 12885_2020_7046_MOESM2_ESM.docx]

|  | Total number of |
| --- | --- |
| Characteristic | Patients (n)% |
| Age (years) | 64.53 ± 0.81 |
| Histological type (n) |  |
| Adenocarcinoma | 12 (27.3) |
| Squamous cell carcinoma | 19 (43.2) |
| Small cell carcinoma | 13 (29.5) |
| Gender (n) |  |
| Men | 25 (56.8) |
| Women | 19 (43.2) |
| Stage Ⅳ (n) | 44 (100.0) |
| Total (n) | 44 (100.0) |

**Supplementary Table 2** The basic information of 44 lung cancer patients for prospective validation analysis.
