# Supplementary material for: Establishment of a serological molecular model for the early diagnosis and progression monitoring of bone metastasis in lung cancer
Source: BMC Cancer. 2020 Jun 16;20:562. doi: 10.1186/s12885-020-07046-2 (PMC7298761; doi:10.1186/s12885-020-07046-2)
Supplement: Supplementary file 3 — Additional file 3: Supplementary Table 3. Correlations of serum levels for BME cytokines and bone turnover markers in 205 patients with primary lung cancer stage IV. [file 12885_2020_7046_MOESM3_ESM.docx]

| Maker 1 | Maker 2 | Correlation Coefficient (r) | *P* value |
| --- | --- | --- | --- |
| BME cytokine |  |  |  |
| CaN | PTHrP | 0.674 | <0.001 |
| OPG | PTHrP | 0.349 | <0.001 |
| IL-6 | PTHrP | 0.294 | <0.001 |
| OPG | CaN | 0.379 | <0.001 |
| IL-6 | CaN | 0.289 | <0.001 |
| Bone turnover maker |  |  |  |
| tP1NP | PTHrP | 0.428 | <0.001 |
| β-CTx | PTHrP | 0.356 | <0.001 |

**Supplementary Table 3** Correlations of serum levels for BME cytokines and bone turnover markers in 205 patients with primary lung cancer stage Ⅳ.
