# Supplementary material for: Establishment of a serological molecular model for the early diagnosis and progression monitoring of bone metastasis in lung cancer
Source: BMC Cancer. 2020 Jun 16;20:562. doi: 10.1186/s12885-020-07046-2 (PMC7298761; doi:10.1186/s12885-020-07046-2)
Supplement: Supplementary file 4 — Additional file 4: Supplementary Table 4. Univariate and multivariate analysis of diagnostic risk factors associated with bone metastasis diagnosis in 205 patients with lung cancer. [file 12885_2020_7046_MOESM4_ESM.docx]

**Supplementary Table 4** Univariate and multivariate analysis of diagnostic risk factors associated with bone metastasis diagnosis in 205 patients with lung cancer.

| Variable | Univariate analysis | | | Multivariate analysis | | |
| --- | --- | --- | --- | --- | --- | --- |
|  | OR | 95% CI | *P value* | OR | 95% CI | *P value* |
| Age |  |  |  |  |  |  |
| ≥ 65yr | 1 | NA | NA |  |  |  |
| < 65yr | 1.66 | 0.94 -2.93 | 0.083 | 2.19 | 0.77-6.28 | 0.144 |
| Gender |  |  |  |  |  |  |
| Women | 1 | NA | NA |  |  |  |
| Men | 0.83 | 0.44 -1.56 | 0.563 | NA | NA | NA |
| Histological type |  |  |  |  |  |  |
| Non-adenocarcinoma | 1 | NA | NA |  |  |  |
| Adenocarcinoma | 2.51 | 1.38 -4.51 | 0.002* | 2.52 | 0.86 -7.38 | 0.092 |
| CaN level |  |  |  |  |  |  |
| ≤1257.8 ng/mL | 1 | NA | NA |  |  |  |
| >1257.8 ng/mL | 8.4 | 4.35-15.23 | <0.001* | 2.34 | 0.82 -6.70 | 0.114 |
| OPG level |  |  |  |  |  |  |
| ≤1547.0 ng/mL | 1 | NA | NA |  |  |  |
| >1547.0 ng/mL | 9.25 | 4.66-18.38 | <0.001* | 6.18 | 2.22-17.22 | <0.001* |
| PTHrP level |  |  |  |  |  |  |
| ≤ 322.3 pg/mL | 1 | NA | NA |  |  |  |
| > 322.3 pg/mL | 20.09 | 9.71-41.56 | <0.001* | 8.34 | 2.81-24.74 | <0.001* |
| IL-6 level |  |  |  |  |  |  |
| ≤14.36 pg/mL | 1 | NA | NA |  |  |  |
| >14.36 pg/mL | 3.23 | 1.79 -5.84 | <0.001* | 1.32 | 0.47 -3.68 | 0.596 |
| tP1NP level |  |  |  |  |  |  |
| ≤ 93.59 μg/L | 1 | NA | NA |  |  |  |
| > 93.59 μg/L | 9.6 | 5.00-18.49 | <0.001* | 7.06 | 2.18-22.92 | 0.001* |
| β-CTx level |  |  |  |  |  |  |
| ≤ 619.7 ng/L | 1 | NA | NA |  |  |  |
| > 619.7 ng/L | 17.76 | 8.63-36.53 | <0.001* | 8.2 | 2.51-26.85 | <0.001* |
| AUC = area under the curve, ROC = receiver operating characteristics, CI = confidence interval, OR = odds ratio, NA= not applicable, * = P < 0.05 was considered statistically significant. | | | | | | |
